# The role of surgical flap design (minimally invasive flap vs. extended flap with papilla preservation) on the healing of intrabony defects treated with an enamel matrix derivative: a 12-month two-center randomized controlled clinical trial

**DOI:** 10.1007/s00784-021-04155-5

**Published:** 2021-09-07

**Authors:** Peter Windisch, Vincenzo Iorio-Siciliano, Daniel Palkovics, Luca Ramaglia, Andrea Blasi, Anton Sculean

**Affiliations:** 1 Department of Periodontology, Semmelweis University, Szentkiralyi str.47.4th floor, 1088 Budapest, Hungary; 2 Department of Periodontology, University of Naples Federico II, Via S. Pansini 5 80131, Naples, Italy; 3 Department of Periodontology, School of Dental Medicine, University of Bern, Freiburgstrasse 7, CH-3010 Bern, Switzerland

**Keywords:** Periodontal disease, Randomized controlled trial, Wound healing, Enamel matrix derivative, Intrabony defects, Surgical periodontal therapy, Minimally invasive flaps

## Abstract

**Objectives:**

Minimally invasive flap designs have been introduced to enhance blood clot stability and support wound healing. Limited data appear to suggest, that in intrabony defects, better clinical outcomes can be achieved by means of minimally invasive flap compared to more extended flaps. The aim of this study was to evaluate the healing of intrabony defects treated with either minimally invasive surgical flaps or with modified or simplified papilla preservation techniques in conjunction with the application of an enamel matrix derivative (EMD).

**Materials and methods:**

Forty-seven subjects were randomly assigned to either test (*N* = 23) or control (*N* = 24) procedures. In the test group, the intrabony defects were accessed by means of either minimally invasive surgical technique (MIST) or modified minimally invasive surgical technique (M-MIST) according to the defect localization while the defects in the control group were treated with either the modified or simplified papilla preservation (MPP) or the simplified papilla preservation technique (SPP). EMD was used as regenerative material in all defects. The following clinical parameters were recorded at baseline and after 12 months: full-mouth plaque score (FMPS), full-mouth bleeding score (FMBS), probing depths (PD), clinical attachment level (CAL), and gingival recession (GR). Early healing index (EHI) score was assessed in both groups 1 week following the surgery. CAL gain was set as primary outcome.

**Results:**

After 12 months follow-up, the CAL gain was 4.09 ± 1.68 mm in test group and 3.79 ± 1.67 mm in control group, while the PD reduction was 4.52 ± 1.34 mm and 4.04 ± 1.62 mm for test and control sites. In both groups, a minimal GR increase (0.35 ± 1.11 mm and 0.25 ± 1.03 mm) was noted. No residual PDs ≥ 6 mm were recorded in both groups. CAL gains of 4–5 mm were achieved in 30.4% and in 29.2% of test and control group, respectively. Moreover, CAL gains ≥ 6 mm were recorded in 21.7% of experimental sites and in 20.8% of control sites. No statistically significant differences in any of the evaluated parameters were found between the test and control procedures (*P* > 0.05). After 1 week post-surgery, a statistically significant difference (*P* < 0.05) between the groups was found in terms of EHI score.

**Conclusions:**

Within the limits of this pilot RCT, the results have failed to show any differences in the measured parameters following treatment of intrabony defects with EMD, irrespective of the employed surgical technique.

**Clinical relevance:**

In intrabony defects, the application of EMD in conjunction with either MIST/M-MIST or M-PPT/SPPT resulted in substantial clinical improvements.

**Supplementary Information:**

The online version contains supplementary material available at 10.1007/s00784-021-04155-5.

## Introduction

Regenerative periodontal therapy aims at reconstructing the lost periodontal structures caused by periodontal disease or trauma and is histologically characterized by formation of cementum with inserting collagen fibers, periodontal ligament, and bone [1]. This healing would lead to the resolution or reduction of the intrabony defect component and of probing depths, gain of clinical attachment level, and minimize soft tissues recession [2]. A number of clinical factors such as patient compliance, morphology of the intrabony defects, regenerative materials (i.e., GTR, EMD, and combination of EMD/bone graft), and surgical flap management have been shown to decisively influence the clinical outcomes following regenerative periodontal therapy [3–7]. Substantial evidence also indicates that blood clot stability plays a critical role during the healing process of intrabony defects [8]. Histological evidence [9] has shown that during the early healing phase, a fibrin clot is formed immediately upon wound closure in the interface between the surgical flap and the root surface. The lack of surgical flap stability may result in the detachment of the fibrin clot followed by apical migration of epithelial cells leading to formation of a long junctional epithelium and no or limited periodontal regeneration. Therefore, would closure enabling primary intention healing is considered a prerequisite to stabilize the blood clot and to support a healing process that facilitates periodontal regeneration.

In traditional periodontal access, flap procedures flap dehiscence occurs most commonly in the interdental area [10, 11]. Therefore, in order to achieve primary closure, several authors suggested to displace the interdental incisions to either vestibularly or orally to preserve the integrity of the papilla (i.e., so-called papilla preservation procedures) [12–15]. However, despite the fact that by using papilla preservation approaches, primary closure of the interdental spaces can be predictably achieved, the flaps are elevated both vestibularly and orally to enable an adequate access to the intrabony defect and adjacent root surfaces. A potential drawback of these surgical approaches is the possible increase in flap mobility, which may negatively influence blood clot stability during the crucial phase of early wound healing.

As biomaterials also developed over time, and regeneration of intrabony periodontal defects was possible to achieve without the application of a barrier membrane, extended flap elevation on both the oral and vestibular aspects could be avoided, thus leading to the development of minimally invasive approaches [16]. For these reasons, based on their papilla preservation technique [14, 15], Cortellini and Tonetti suggested to limit the incision and mucoperiosteal flap elevation at the defects involved interdental area alone without intrasulcular incisions extending to the midline of adjacent teeth (minimally invasive surgical technique, MIST) [17]. Subsequently, the MIST approach was modified and the papilla was elevated at the buccal aspect alone (modified minimally invasive surgical technique, M-MIST) in order to additionally improve wound stability [18]. Likewise, Trombelli and co-workers purposed a similar flap design in order to access at the intrabony defects from only one side; however, flap elevation was determined by the localization of the defect and mesiodistal extension is not limited (single-flap approach, SFA) [19].

These surgical techniques, in conjunction with an enamel matrix derivative (EMD) [20] alone or combined with bone grafts [21], yielded substantial clinical improvements evidenced by probing depth reduction and clinical attachment gain. In addition, minimally invasive surgical techniques seem to offer improved outcomes in terms of invasiveness reduction, post-surgical complications, and patient morbidity [22].

These clinical findings were also supported by an animal study published by Azuma and co-workers on the healing mechanisms of periodontal intrabony defects [23]. Histological and immunohistochemistry analysis demonstrated an accelerated shift from blood clots to granulation tissue and an earlier, more significant increase of type III collagen at surgical sites treated with single-flap periodontal surgery compared to conventional flap elevation, resulting in a more efficient wound healing.

Although clinical trials report encouraging outcomes following treatment of intrabony defects by means of minimally invasive surgical approaches and use of regenerative materials [24], the superiority of these techniques compared to more extended papilla preservation flaps (i.e., modified papilla preservation flap, M-PPT, or simplified papilla preservation flap, SPPT) remains to be determined. A very recent systematic review [25] reported that the efficacy of minimally invasive surgeries for periodontal regenerative therapy cannot be verified due to the lack of studies comparing these techniques. Hence, the aim of present study was to evaluate the healing of intrabony defects (in terms of clinical parameters, i.e., CAL gain, PD reduction, and GR change) treated with either minimally invasive surgical flap or extended flap with papilla preservation technique in conjunction with the application of an enamel matrix derivative (EMD).

## Materials and methods

### Ethical statements

The research protocol (N° 0,012,240) was submitted and approved by the Institutional Review Board (IRB) of the University of Naples Federico II, Italy (Approval Number: 103/17) and by the Institutional Review Board (IRB) of the Semmelweis University, Budapest, Hungary (Approval Number: 195/2017). All patients received a copy of the research protocol and signed an informed consent. The patients were informed that they were free not to continue the trial at any time. The study was conducted in accordance with the Helsinki Declaration of 1975, as revised in 2013 [26].

### Study design

The study was designed as double-center, randomized controlled, superiority clinical trial.

Since no previous studies have compared the performance of minimally invasive surgical flaps with respect to extended flaps with papilla preservation in the periodontal regeneration of intrabony defects treated using EMD, the present investigation was considered as a pilot study. Patients needing regenerative periodontal therapy by means EMD were randomly allocated in the test or control group. In each patient, one isolated intrabony defect was selected for investigation. In the test group, a minimally invasive surgical flap (either MIST or M-MIST) was performed in order to access the intrabony defects, while in the control group, an access flaps with papilla preservation techniques (modified papilla preservation technique: M-PPT or simplified papilla preservation technique: SPPT) extending to the adjacent teeth were elevated at buccal and oral aspects. The null hypothesis of no statistically significant difference between two surgical procedures was verified. The investigation was performed at two research centers: (i) Department of Periodontology, University of Naples Federico II (Italy) and (ii) the Department of Periodontology, Semmelweis University, Budapest (Hungary). A cooperation agreement for the present study was signed between the two Universities. The study was registered on ClinicalTrial.gov registry (ID: NCT04542746). The present randomized controlled clinical trial was conducted according to CONSORT statement (http://www.consort-statement.org/). CONSORT 2010 checklist is reported in Supplementary Materials Appendix 1.

### Patient selection

All participants were recruited from two academic centers: Department of Periodontology, University of Naples Federico II (Italy) and from the Department of Periodontology, Semmelweis University, Budapest (Hungary). Data were collected from both research centers.

### Eligibility criteria for participants

Inclusion criteria:

- Patients with diagnosis of periodontitis (stage III or IV) [27].
- Male and female.
- Age ≥ 18 years old.
- Single-rooted and multi-rooted teeth in either the maxilla or the mandible.
- Presence of interdental periodontal pocket with PD ≥ 6 mm associated to an intrabony component ranged from 3 to 6 mm.
- Contained intrabony defects (i.e., intrabony defects with ≥ 80% 3-wall component).

Exclusion criteria:

- Patients with systemic diseases.
- Pregnant or lactating.
- Tobacco smokers (≥ 10 cigarettes per day).
- Patients showing a FMPS [28] and FMBS [29] ≥ 25%.
- Multi-rooted teeth with class II and class III furcation defects.
- Third molars.
- Teeth with grade III mobility.

### Interventions

#### Pre-treatment

Non-surgical periodontal treatment was performed through scaling and root planning (SRP) in combination with oral hygiene instructions and motivation. The re-evaluation was made after 6 months and the patients who met the inclusion criteria were included in the study.

#### Surgical procedure

**Fig. 1:**
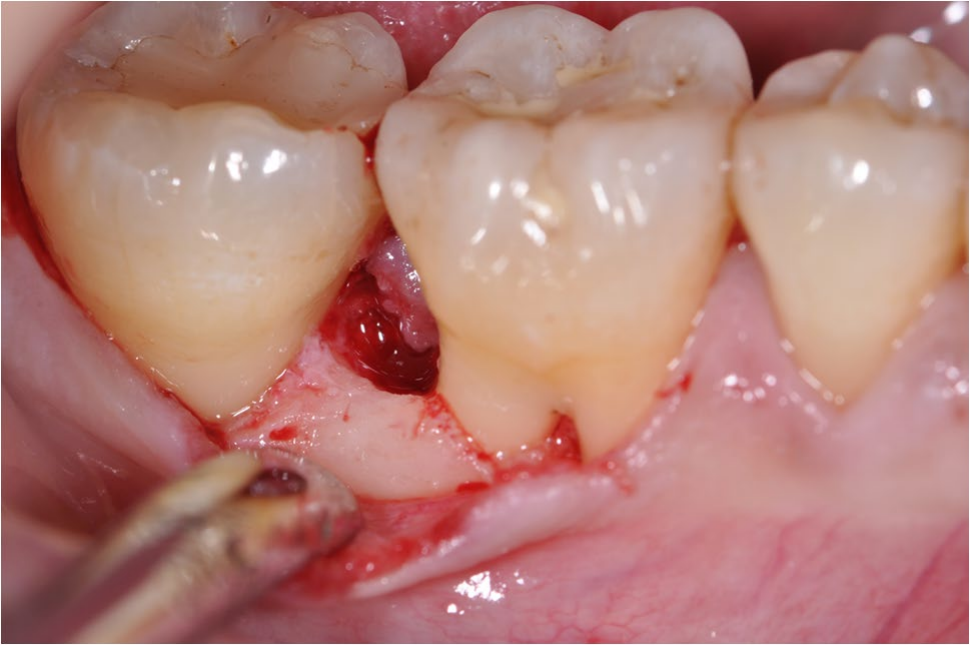
Surgical access to intrabony defect with modified minimally invasive surgical technique (M-MIST)

**Fig. 2:**
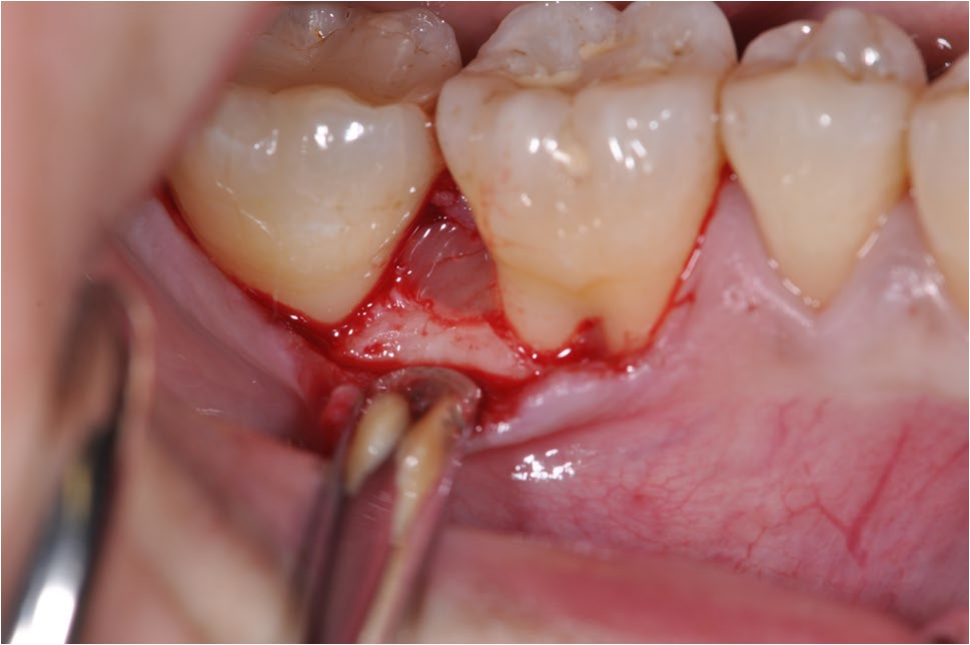
EMD application after root conditioning using 24% EDTA gel

**Fig. 3:**
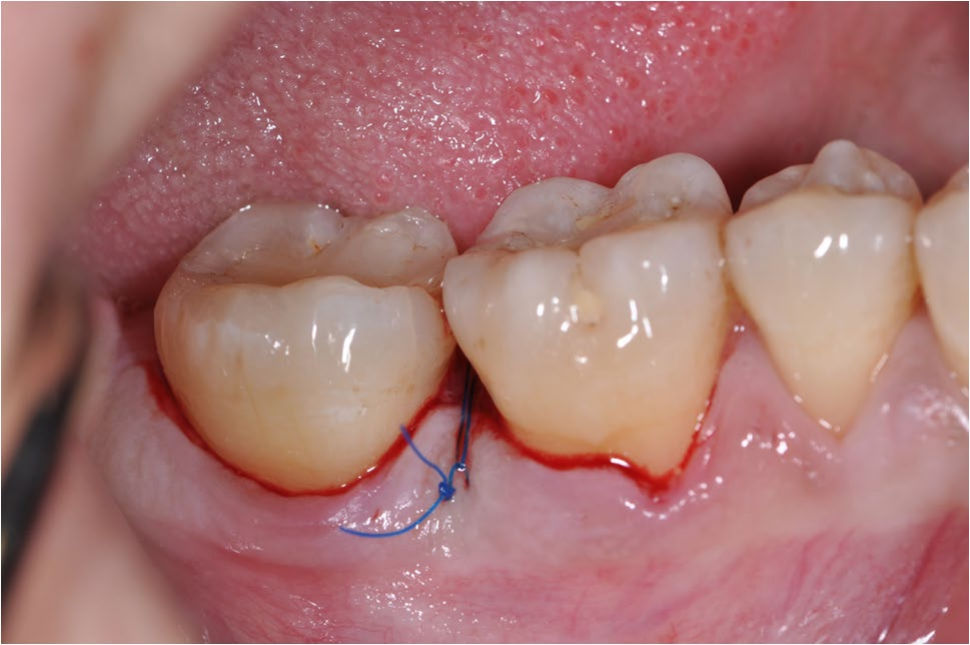
Primary wound closure of interdental area

Prior to surgery, the clinical parameters were recorded and the patients were randomly allocated in the test or control group, respectively. After local anesthesia, pre-surgical bone sounding was performed to determine defect anatomy and location. All surgical procedures were performed by two expert periodontists (VIS, PW) using 4.0 magnifying loupes. Intraosseous defects of test group were treated following the principle of minimally invasive surgical flaps (MIST or M-MIST) as described in previous studies [17, 18]. Briefly, only the defect-associated papilla was accessed and the mucoperiosteal flap was minimally elevated at the buccal and oral aspect (MIST) or at the buccal aspect alone (M-MIST). Whenever the intraosseous defect was cleansable from the buccal aspect, only M-MIST was performed (Fig. 1), while if the intraosseous defect was not cleansable from the buccal side, the MIST was applied [30]. All intrabony defects were accessed from buccal aspect. In the control group, a more extended flap with papilla preservation technique was elevated at buccal and oral aspects. An intrasulcular incision was extended to at least one tooth mesially and distally from the tooth presenting the intraosseous defects. Depending on the mesiodistal width of inter-proximal space, two different techniques were selected to access the intrabony defect area. The MPPT was used at sites with an interdental width > 2 mm, whereas the SPPT was applied at sites with an inter-proximal width < 2 mm [30]. After surgical access and removal of granulation tissue, scaling and root planning by means of Gracey metal mini-curettes and ultrasonic scaler were made in both groups. Subsequently, root conditioning with 24% EDTA gel (PrefGel®, Straumann, Basel, Switzerland) was performed in both groups for 2 min followed by copious rinsing with a sterile saline solution. After root conditioning, EMD (Emdogain®, Straumann, Basel, Switzerland) was placed in all intraosseous defects using a sterile syringe (Fig. 2) [31]. In both groups, primary wound closure was achieved using a 5–0 monofilament non-bioresorbable suture material (Fig. 3).

#### Post-surgical instructions and infection control

**Fig. 4:**
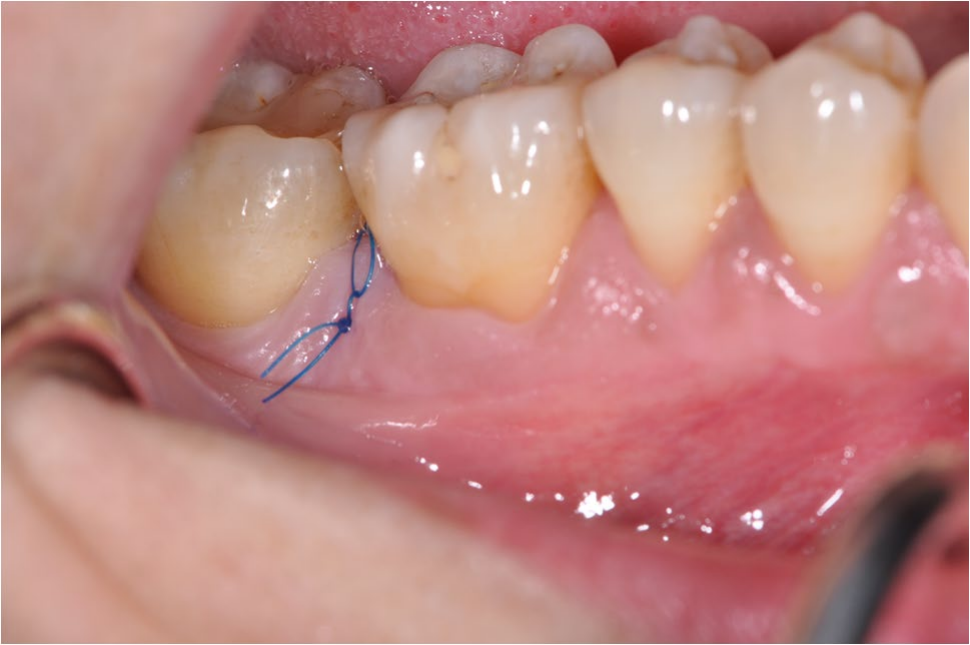
Early wound healing at 1 week

No systemic antibiotics were prescribed. All patients received 600 mg ibuprofen immediately before surgical interventions and after 4 h. All patients were asked not to brush the treated area for 2 weeks and rinse twice daily for 1 min with a 0.12% chlorhexidine-digluconate mouth rinse. Sutures were removed after 7 days (Fig. 4) and all patients were recalled at 2, 3, and 4 weeks and after 3, 6, 9, and 12 months for oral hygiene instructions and motivation and professional supragingival tooth cleaning. At 12 months postoperatively, the final periodontal examination was performed.

### Outcome measures

#### Clinical measurements

The primary outcome variable of the study was to assess the clinical attachment level (CAL) gain. CAL was measured from cemento-enamel junction (CEJ) to the bottom of the pocket.

The secondary outcomes were full-mouth plaque score (FMPS), full-mouth bleeding score (FMBS), probing depth (PD), gingival recession (GR), and early healing index (EHI) [32].

PD was defined as the distance from gingival margin to the bottom of the pocket, while GR was the distance from cemento-enamel junction (CEJ) to gingival margin.

Early healing index (EHI) [32] was based on 5 scores: score 1 (complete flap closure — no fibrin line in the inter-proximal area), score 2 (complete flap closure — fine fibrin line in the inter-proximal area), score 3 (complete flap closure — fibrin clot in the inter-proximal area), score 4 (incomplete flap closure — partial necrosis of the inter-proximal area), and score 5 (incomplete flap closure — complete necrosis of the inter-proximal area). FMPS and FMBS were recorded at six sites per tooth, while PD, CAL, and GR represent the measurements at the deepest site of intraosseous defect of each tooth. All clinical variables were assessed using a graduated manual periodontal probe (PCP-UNC 15®, Hu-Friedy, Chicago, IL, USA) at baseline and after 12-month follow-up. EHI was valuated at 1 week after surgery.

#### Intra-surgical defect characterization

The following intra-surgical parameters were also collected: vertical distance between CEJ to the bottom of the intraosseous defects (CEJ-BD), vertical distance from the bone crest to the bottom of the defect (INTRA), and horizontal distance from root surface to the bone crest (WIDTH) [33, 34].

### Sample size calculation

Sample size was set a priori at 24 patients/group based on the pilot nature of this study.

### Randomization

A computerized random number generator (Random.org;www.random.org) was used in order to random assign the subjects to experimental or control procedures. A simple randomization without restrictions was done. The allocation concealment was made associating even numbers to the test procedure and odd number to the control procedure. The cards with numbers were closed in opaque envelopes and treatment allocation was performed before the surgical treatment by opening the envelope containing the number.

The random allocation sequence was generated by L.R., while the patients were enrolled and assigned to interventions by A.B. in the University of Naples Federico II and D.P. in the University of Budapest.

### Blinding and calibration

All surgical treatments were performed by two expert clinicians (VIS, University of Naples, PW, Semmelweis University Budapest). Periodontal parameters were recorded at baseline and after 12 months by two calibrated examiners (A.B. and D.P.). The examiners and patients were masked with respect to test or control procedures. Examiners attended a training and calibration session on a total of 20 patients not involved in the trial (kappa coefficient agreement = 0.87). The calibration meeting was performed at Department of Periodontology, University of Naples Federico II (Italy).

### Statistical analysis

Statistical analysis was performed using a statistical software package (NCSS-PASS, NCSS, Kaysville, UT) and the patient was considered as statistical unit. Means and standard deviations were calculated for age, FMPS, FMBS, PD, CAL, GR, CEJ-BD, INTRA, and WIDTH. All clinical parameters were expressed in millimeters, while FMPS and FMBS were expressed in percentages. EHI was reported as number of sites. The heterogeneity of the sample with respect to test and control procedures was verified using the Fisher test, while the comparison of proportions of males and females was based on *χ*^2^ test. In terms of heterogeneity, the distribution of intrabony defects in test and control group was tested using Kolmogorov–Smirnov test. Paired *t*-test was performed to compare the outcomes within the two groups; meanwhile, an unpaired *t*-test was used to compare the findings between test and control groups. Mantel–Haenszel *χ*^2^ test was chosen to compare the frequency distribution of CAL changes, residual PDs, and EHI scores between test and control groups. The influence of the center and treatment effects on dependent variable CAL change was calculated using a generalized linear model. FMPS, FMBS, and baseline values of PDs were selected for stratification. A logistic regression model was used to analyze the factors (center, FMPS, FMBS, and flap design) that may statistically significantly affect the probability to gain at least 4 mm CAL. A *P* value < 0.05 was set to accept a statistically significant difference.

## Results

### Participants and recruitment

**Fig. 5:**
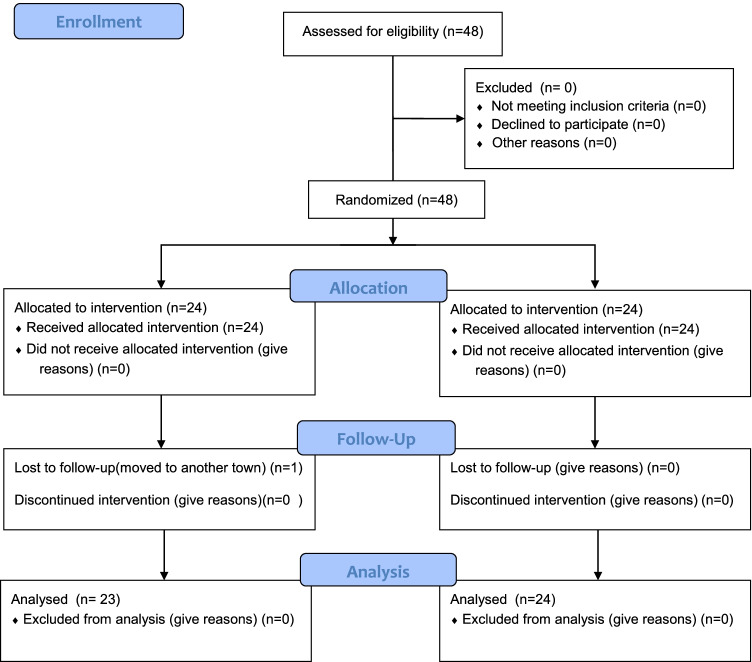
CONSORT flow diagram

According to eligibility criteria, a total of 48 patients with 48 intrabony defects were recruited for the study. One patient (test group) was lost (drop-out) at University of Naples Federico II during the follow-up visits because he moved to another town. After 12 months, a total of 47 patients (23 and 24 patients for test and control groups) have completed the trial. Twenty-nine patients were enrolled in the University of Naples and 18 in the University of Budapest. Figure 5 illustrates the flow chart of the experimental trial.

### Demographic characteristics

Table 1 illustrates the demographic characteristics of patient’s population at baseline. No statistically significant differences were found between the test and control groups (*P* > 0.05) (Table 1).

**Table 1:** Demographic characteristics

	Test group (*N* = 23)	Control group (*N* = 24)	*P* value
Gender (m/f)	9/14	10/14	0.99
Mean age ( years)	48.9 ± 9.7	47.8 ± 5.3	0.90
Smokers (y/n)	2/21	2/22	0.99
Dental arch (max/Mdb)	11/12	16/8	0.24

*M*, male; *f*, female; *y*, yes; *n*, no; *max*, maxilla; *Mdb*, mandible

### FMPS and FMBS changes

FMPS and FMBS are presented in Table 2. At baseline, a FMPS of 17.5 ± 4.2% and 17.2 ± 5.9% were recorded in the test and control group, respectively. After 12 months, the FMPS was 17.3 ± 3.8% in the test group and 16.8 ± 5.8% in the control group, respectively. At baseline, the FMBS measured 14.5 ± 5.8% in the test and 16.0 ± 5.2% in the control group, respectively. At 12 months, mean FMBS measured 14.1 ± 5.5% in the test and 15.3 ± 4.5% in the control group, respectively. No statistically significant differences were found between baseline and follow-up examination in each group (*P* > 0.05) and no statistically significant differences were recorded between the groups (*P* > 0.05) (Table 2).

**Table 2:** FMPS and FMBS changes at baseline and after 12 months follow-up

Parameters	Test group (*N* = 23)	Control group (*N* = 24)	*P* value
***FMPS (%)***			
Baseline	17.5 ± 4.2	17.2 ± 5.9	0.81
1 year	17.3 ± 3.8	16.8 ± 5.8	0.35
Change	0.2 ± 1.1	0.4 ± 1.1	0.13
*P* value	0.73	0.43	
***FMBS (%)***			
Baseline	14.5 ± 5.8	16.0 ± 5.2	0.38
1 year	14.1 ± 5.5	15.3 ± 4.5	0.11
Change	0.4 ± 1.3	0.7 ± 2.2	0.13
*P* value	0.62	0.61	

### Intrabony defects characteristics

Table 3 shows the characteristics of the intrabony defects after surgical flap elevation and granulation tissue removal. The CEJ-BD measured 10.22 ± 2.28 mm in the test and 9.54 ± 2.27 mm in the control defects, respectively. The defects in test group revealed an intrabony component (INTRA) of 4.39 ± 1.20 mm, while the corresponding value measured 4.63 ± 1.55 mm in the control group. The WIDTH of the intrabony defects measured 3.61 ± 1.03 mm in the test group and 3.61 ± 0.91 mm in the control group, respectively. No statistically significant differences were found between the 2 groups (*P* > 0.05) (Table 3).

**Table 3:** Intrabony defects characteristics

	Test group (*N* = 23)	Control group (*N* = 24)	*P* value
CEJ-BD (mm)	10.22 ± 2.28	9.54 ± 2.27	0.31
INTRA (mm)	4.39 ± 1.20	4.63 ± 1.55	0.57
WIDTH (mm)	3.61 ± 1.03	3.61 ± 0.91	0.73

### Changes in probing depth, clinical attachment level, and gingival recession

Changes in PD, CAL, and GR are reported in Table 4. A statistically significant PD reduction (*P* < 0.05) was noted in each group between baseline and after 12 months follow-up period. Mean PD decreased from 7.22 ± 1.17 to 2.78 ± 0.74 mm in test group and from 7.25 ± 1.39 to 3.21 ± 0.83 mm in the control group. Mean PD reduction was 4.52 ± 1.34 mm and 4.04 ± 1.62 mm in the test and control groups, respectively. No statistically significant differences (*P* > 0.05) were recorded between the 2 groups at 12 months follow-up. In each group, a statistically significant CAL change (primary outcome) was found between baseline (i.e., test group 8.82 ± 1.92 mm and control group 8.71 ± 2.29 mm) and after 12 months (i.e., test group 4.78 ± 2.09 mm and control group 4.92 ± 1.59 mm) (*P* < 0.05). After 12 months, the intrabony defects treated with the test procedure showed a mean CAL gain of 4.09 ± 1.68 mm, while in the control sites, the corresponding value amounted to 3.79 ± 1.67 mm. No statistically significant difference was found in terms of CAL gain between the two groups (*P* > 0.05). No statistically significant changes (*P* > 0.05) were noted in terms of mean GR at baseline (i.e., 1.65 ± 1.23 mm in the test group versus 1.46 ± 1.25 mm in the control group) and after 12 months (i.e., 2.00 ± 1.88 mm in the test group and 1.71 ± 1.60 mm in the control group) in each group. After 12 months, the intra-group comparison failed to reveal a statistically significant difference in terms of GR increase (0.35 ± 1.11 mm vs. 0.25 ± 1.03 mm) (*P* > 0.05) (Table 4).

**Table 4:** Change in PD, CAL, and REC after 12 months follow-up

	Test group (*N* = 23)	Control group (*N* = 24)	*P* value
PD (mm)			
Baseline	7.22 ± 1.17	7.25 ± 1.39	0.93
1 year	2.78 ± 0.74	3.21 ± 0.83	0.07
Changes	4.52 ± 1.34	4.04 ± 1.62	0.28
*P* value	0.001	0.001	
CAL (mm)			
Baseline	8.82 ± 1.92	8.71 ± 2.29	0.85
1 year	4.78 ± 2.09	4.92 ± 1.59	0.80
Changes	4.09 ± 1.68	3.79 ± 1.67	0.55
*P* value	0.001	0.001	
GR (mm)			
Baseline	1.65 ± 1.23	1.46 ± 1.25	0.68
1 year	2.00 ± 1.88	1.71 ± 1.60	0.46
Changes	0.35 ± 1.11	0.25 ± 1.03	0.76
*P* value	0.15	0.25	

### Frequency distribution of residual PDs and CAL changes

No statistically significant differences were found between the test and control groups in terms of residual PDs and CAL changes at 12 months follow-up (*P* > 0.05). No residual PDs ≥ 6 mm were detected in both groups. A CAL gain of 0–1 mm was obtained in 4.3% in the test group and in 8.3% in the control group, respectively. CAL gains of 2–3 mm were measured in 43.5% of the test and in 41.7% of the control defects. CAL gains of 4–5 mm were measured in 30.4% of the experimental sites and 29.2% of the control sites, while in 21.7% of the test sites and in 20.8% of the control sites, a CAL gain ≥ 6 mm was obtained (Table 5).

**Table 5:** Frequency distribution (%) of residual PD and CAL changes after 12 months follow-up

	Test group (*N* = 23)	Control group (*N* = 24)	*P* value
Residual PD (mm)			
0–1	0	0	0.28
2–3	87.0	70.8	
4–5	13.0	29.2	
≥ 6	0	0	
CAL changes (mm)			
0–1	4.3	8.3	0.96
2–3	43.5	41.7	
4–5	30.4	29.2	
≥ 6	21.7	20.8	

### Early healing index

Table 6 summarizes the early healing index (EHI) at 1 week after therapy. A statistically significant difference was recorded between the groups (*P* < 0.05). Complete flap closure was observed in 22 sites of test group and 23 sites of control group. The number of sites showing an optimal wound healing (score 1) was 17 in the test group and 10 in the control group, respectively. In one site of the control group was observed an incomplete flap closure with partial necrosis of the inter-proximal area (score 4), while one site from the test group showed an incomplete flap closure and complete necrosis of the inter-proximal area was noted (score 5) (Table 6).

**Table 6:** Frequency distribution (number of sites) of EHI at 1 week after treatment

	Overall	
EHI score	Test group	Control group
1	17	10
2	3	9
3	2	4
4	0	1
5	1	0
*P* value	0.02	

### Center effect on CAL gain

Table 7 reports the center effect on the variable CAL gain. An estimate of 4.44 ± 0.77 was recorded for the center, while an estimate of 0.30 ± 0.98 was observed for the treatment. Estimates of 6.00 ± 1.52 and 4.33 ± 1.85 were reported for FMPS and FMBS, respectively. Finally, an estimate for PD at baseline was 4.33 ± 2.01. No center effect was found (Table 7).

**Table 7:** Center effect for the clinical attachment level changes

Parameter	Estimate	*P* value
Center	4.44 ± 0.77	0.099
Treatment	0.30 ± 0.98	0.548
FMPS	6.00 ± 1.52	0.332
FMBS	4.33 ± 1.85	0.218
PD baseline	4.33 ± 2.01	0.621

### *Factors affecting the probability to obtain a CAL gain* ≥ *4 mm*

Table 8 illustrates the logistic regression analysis of factors affecting the treatment outcomes. Center (*OR* = 0.91; C.I. 0.65–1.27), FMPS (*OR* = 1.03; C.I. 0.77–1.37), FMBS (*OR* = 1.55; C.I. 0.15–16.27), and flap design (*OR* = 0.86; C.I. 0.24–3.10) did not affect statistically significantly the probability to obtain a CAL gain ≥ 4 mm or more (*P* > 0.05) (Table 8).

**Table 8:** Logistic regression analysis of parameters significantly influencing the probability to achieve a CAL gains ≥ 4 mm

Parameter	*P* value	Odds ratio	95% C.I
Center	0.57	0.91	0.65–1.27
FMPS	0.86	1.03	0.77–1.37
FMBS	0.71	1.55	0.15–16.27
Flap design	0.82	0.86	0.24–3.10

## Discussion

The main objective of the present study was to compare the efficacy of minimally invasive surgical flaps (MIST/M-MIST) with the more extended papilla preservation flaps on the healing of intrabony defects treated with EMD. In order to avoid treatment bias, the intrabony defects in both groups received exactly the same regenerative material (i.e., EMD). Since minimally invasive flaps are based on reduced surgical access in the interdental area, the application of a membrane alone or combined with bone grafts is impossible [35, 36]. For this reason, other regenerative procedures such as GTR were excluded, despite the fact that the intrabony defects of control group were treated by means of either modified papilla preservation technique or simplified papilla preservation technique, respectively, where the flaps are elevated at both buccal and oral aspects. Although the use of EMD in combination with a minimally invasive surgical flap (i.e., M-MIST) is applicable in any defect anatomy [30], it has been reported that contained 3-wall intrabony defects treated with papilla preservation flaps and EMD yielded a 269% higher chance than 1-wall defects to gain 3 mm CAL or more [37]. In addition, in the most non-contained intrabony defects (79.3%) treated by means of EMD and papilla preservation flaps, a residual PD ≥ 6 mm was recorded after 12 months follow-up [38]. For these reasons, non-contained intrabony defects were excluded from the present study. Furthermore, the test and control procedures were applied in periodontal pockets associated with intrabony components ranging from 3 to 6 mm. In fact, the presence of deep intrabony defects (i.e., INTRA > 6 mm) would have required a more extended flap also in the test group in order to provide an adequate visibility for instrumentation [30]. The CAL gains recorded at intrabony defects treated by means of minimally invasive flaps and EMD (4.09 ± 1.68 mm) are similar to those reported in other studies [39, 40]. However, this result is in contrast to Riberio and co-workers [36] who have reported a CAL gain of 3 mm at sites accessed with minimally invasive surgery. The reason of the lower CAL gain reported by Riberio and co-workers [36] may be related to defect selection since in that study the morphology of the intrabony defects was not provided. Thus, it cannot be excluded that at least a part of the periodontal pockets selected for the therapy with EMD were associated with non-contained intrabony defects. In the present study, statistically significant CAL gain and PD reduction were measured in each group at 12 months, but no statistically significant differences were recorded between the two groups. These results are in agreement with previously reported findings on the healing of intrabony defects accessed with SFA or double extended flap with papilla preservation technique in conjunction with recombinant platelet derived growth factor (rh-PDGF-BB) and beta tricalcium phosphate (ß-TCP) [41]. The authors found no statistically significant differences between the surgical procedures in any of the investigated clinical parameters (i.e., CAL gain of 4 mm vs. 3.2 mm and PD reduction 4.1 mm vs. 3.6 mm). However, the patients treated with SFA reported lower pain in the first postoperative days compared to those receiving the more extended flaps. In the present trial, at 12 months, both treatments resulted in statistically significant PD reduction without residual sites with PD ≥ 6 mm. Likewise, a minimal gingival recession increase was noted in both groups. In agreement with a previous study [41], the small increase in gingival recession for the control sites may have been explained by the passive coronal displacement of the vestibularly extended flap during suturing. At 1 week after therapy, a statistically significant difference was recorded in terms of EHI score between test and control procedures. The EHI score may be influenced by the choice of the suturing material (5–0 monofilament suture), since it may be hypothesized that 5–0 monofilament sutures have a higher tendency to compress the wound, which, in turn, might have influenced the healing. Usually, when MIST or M-MIST is applied, wound closure is ensured with 6–0 or 7–0 sutures [17, 18]. However, it needs to be kept in mind that at present there is insufficient evidence demonstrating a negative influence on the clinical outcomes obtained in intrabony defects following regenerative surgery as related to the size of the sutures.

The small differences recorded between test and control procedures can be explained by the surgical flap design in the test group. The MPP and SPP were originally proposed for membrane application and for this reason, the margin of residual bone crest was exposed for about 2–3 mm. The fact that such a mobilization is not any longer necessary when EMD is used, has led to the development of MIST and M-MIST. Compared to MIST and M-MIST, MPP and SPP are mobilized not only to the adjacent mesial and distal teeth but also apically across the mucogingival line to facilitate coronal reposition. Thus, the original MPP and SPP cause substantially more instability than the approach that is called MPP and SPP in this study. It may thus be assumed that for the abovementioned reasons, the small differences between the surgical techniques used in test and control group did not result in statistically significant differences. The difference obtained for the primary outcome (CAL gain) between test and control procedures was less than 1 mm. From a clinical point of view, a difference less than 1 mm is not clinically relevant and therefore, it does not scientifically justify the advantage of one over the other technique.

A limitation of the present study was the impossibility to calculate an adequate sample size, since no previous studies have compared the clinical outcomes obtained in intrabony defects treated with EMD and accessed with either minimally invasive flaps or M-PPT or SPPT. Until now, the majority of the available RCTs have only compared the healing of intrabony defects treated by means of a minimally invasive flap and a regenerative material (i.e., either EMD or growth factors) with a minimally invasive flap alone [39, 42]. However, in those studies, the main objective was to evaluate the role of the regenerative material and not the performance of the surgical technique (i.e., the influence of the surgical flap). Therefore, the present RCT was considered as a pilot study and the results need to be validated in further adequately powered studies. In the present study, no pressure-calibrated probes and splints were used to assure identical probing location pre- and post-surgically. While on one hand, this might be considered a limitation of the study; on the other hand, all measurements were made by experienced and calibrated periodontists. The calibration sessions included a total of 20 patients who were not included in the trial yielding a kappa coefficient agreement of 0.87.

## Conclusions

Within the limits of this pilot RCT, the results have failed to show any differences in the measured parameters following treatment of intrabony defects and EMD, irrespective of the employed surgical technique.

## Supplementary Information

Below is the link to the electronic supplementary material.

Supplementary file1 (DOC 219 KB)
